# *In Vivo* Pharmacological and Anti-inflammatory Evaluation of Xerophyte *Plantago sempervirens* Crantz

**DOI:** 10.1155/2019/5049643

**Published:** 2019-06-02

**Authors:** Anca D. Farcaș, Augustin C. Moț, Alina E. Pârvu, Vlad Al. Toma, Mirel A. Popa, Maria C. Mihai, Bogdan Sevastre, Ioana Roman, Laurian Vlase, Marcel Pârvu

**Affiliations:** ^1^Department of Biology, Faculty of Biology and Geology, Babeș-Bolyai University, Cluj-Napoca RO-400028, Romania; ^2^Department of Biomolecular Physics, National Institute for Research and Development of Isotopic and Molecular Technologies, Cluj-Napoca RO-400293, Romania; ^3^Department of Chemistry, Faculty of Chemistry and Chemical Engineering, Babeș-Bolyai University, Cluj-Napoca RO-400028, Romania; ^4^Department of Pathophysiology, Faculty of Medicine, “Iuliu Hațieganu” University of Pharmacy and Medicine, Cluj-Napoca RO-400012, Romania; ^5^Department of Regenerative Medicine, Institute of Cellular Biology and Pathology “Nicolae Simionescu”, Bucharest RO-0500568, Romania; ^6^Department of Pathophysiology, Faculty of Veterinary Medicine, University of Agricultural Sciences and Veterinary Medicine, Cluj-Napoca RO-400372, Romania; ^7^Department of Experimental Biology and Biochemistry, Institute of Biological Research, Cluj-Napoca, Branch of NIRDSB, Bucharest RO-400115, Romania; ^8^Department of Pharmacy, Faculty of Pharmacy, “Iuliu Hațieganu” University of Pharmacy and Medicine, Cluj-Napoca RO-400012, Romania

## Abstract

Known for centuries throughout the world, *Plantago* species have long been used as traditional herbal remedies for many diseases related to inflammatory conditions of the skin, respiratory and digestive tract, or even malignancy. This study is aimed first at investigating the *in vitro* antioxidant and regenerative effects of *Plantago sempervirens* Crantz hydroalcoholic extract followed by an *in vivo* experiment using a turpentine oil-induced inflammation model. The *in vitro* evaluation for antioxidant activity was performed using classical assays such as DPPH and TEAC scavenging assays but also EPR, and the total phenolic content was determined using the Folin-Ciocalteu reagent. The wound healing assay was performed on human cells (Human EA.hy926). Besides, the prooxidant activity was determined using a method which involves *in situ* free radical generation by laccase and the oxidation of haemoglobin. On turpentine oil-induced inflammation in rats, the *in vivo* effects of three doses of *P. sempervirens* extracts (100%, 50%, and 25%) were assessed by measuring oxidative stress (MDA, TOS, OSI, NO, CAT, and SOD) and inflammatory (CRP, WBC, and NEU) parameters. Having a rich polyphenolic content, the xerophyte *P. sempervirens* exhibited a strong *in vitro* antioxidant activity by scavenging radicals, enhancing cell regeneration, and reducing oxidative stress markers. Diluted *P. sempervirens* extract (25%) exhibited the best antioxidant, wound healing, and anti-inflammatory activity.

## 1. Introduction

*Plantago* herbs are known throughout the world as old medicinal plants, but their medical potential is still underestimated. In the first century AD, Greek physicians depicted the wound healing potential of well-known *P. major*. In Asia and Europe, the pharmacological studies have shown that the aerial parts of these plants are still used as natural remedies for several skin, respiratory, digestive, and reproduction organ diseases, involving an inflammatory response or cancer [1–4]. Nowadays, there is an upsurge of interest in the therapeutic potential of medicinal plants, mostly because of their prophylactic or therapeutic efficiency, low toxicity, and side effects as compared with the synthetic conventional drugs. Due to their high fiber content, in many countries, *Plantago* herbs are commonly added in food products, such as salads, teas, yoghurts, and soups. In cattle, grazing on *P. lanceolata*-rich pastures enhances the general health status and reduces the need for antibiotic therapy [5]. The most renowned *Plantago* species that have crossed the traditional medicine boundaries are still used in modern medicine: *P. major*, *P. lanceolata*, *P. media*, *P. indica*, *P. ovata*, and *P. asiatica* [6]. *P. sempervirens* is a less common *Plantago* species, a typical xerophyte found in barren hills and low mountain pastures, in West Europe, and there are few studies concerning its chemical composition [7], but there is still an intriguing lack of information about its properties and medical potential. In Romania, *P. sempervirens* is also found in spontaneous flora, near Targu-Mures, a city in Transylvania [8].

*Plantago* is a genus comprising more than 200 plant species used extensively all over the world as functional foods and remedies for a wide range of diseases [4, 9]. The *Plantago* genus is a promising source of novel bioactive molecules and multifunctional polysaccharides because only a few species have been investigated comprehensively thus far [9]. In Romania, numerous studies regarding *Plantago* species are focused on herbaceous perennial plants as *Plantago major* L., *Plantago lanceolata*, and *Plantago media* L., from which the leaves are mostly used. *Plantago major* L. (common plantain) is an herbaceous plant with a basal rosette of leaves. Each leaf has an elliptical to ovate shape, an acute apex, and five to nine prominent veins. Petiole length is equalling with or slightly longer/rarely shorter than the lamina [10]. The flowers are small, are greenish-brown, and form a dense spike in the top of a stem. *Plantago lanceolata* L. (English plantain, narrow-leaved plantain) has lanceolate basal leaves with 5-7 parallel veins. Petiole length is 1/3 or equalling with the length of the lamina [10]. The flowers are small, are green-brownish, and form an ovoid inflorescence in the top of a stem. *Plantago media* L. (hoary plantain) is a perennial plant with a basal rosette of leaves which are ovate to elliptical and have 7-9 veins. Lamina trichomes are on both epidermis and are curly, abundant, or sparsely scattered [10]. Petiole length is shorter than the leaf lamina [10]. The greenish-white flowers have long pink-purple stamens and form a spike on the top of the stem.

One of the modern medicine perspectives is the use of antioxidants in order to maintain cellular normal redox balance. The antioxidants are chemical compounds known for the ability to scavenge reactive radicals, such as reactive oxygen species (ROS) and reactive nitrogen species (RNS) [11]. Thereby, there is particular interest in the study of new sources of natural antioxidants, which have less toxicity and may be useful adjuvants in health and diseases. Some *Plantago* species have been already investigated for their antioxidant capacity [1, 2, 5, 12]. For a better understanding of the antioxidant potential, the plant extracts have to be tested both *in vitro* and *in vivo*.

Moreover, because oxidative stress is associated with an inflammatory response [13, 14], the anti-inflammatory effects have to be tested as well. The endogenous antioxidant defence comprises enzymatic and nonenzymatic mechanisms [15]. Plant-based compounds may be potential sources of exogenous adjuvant antioxidants [16]. Therefore, our paper is aimed at making an ethnopharmacological investigation *P. sempervirens*, regarding its phytochemical analysis, antioxidant and prooxidant activity, and *in vitro* wound healing properties. Antioxidant and anti-inflammatory effects were further tested *in vivo* on an experimental acute inflammation.

## 2. Materials and Methods

### 2.1. Plant Material and Extract Preparation

The aerial parts of *P. sempervirens* were collected from the Botanical Garden “Alexandru Borza” in Cluj-Napoca, Romania, and were identified at the Herbarium of the Botanical Garden, where a voucher specimen was deposited (*P. sempervirens*—CL663373). The *Plantago* extract was prepared by the percolation method at room temperature for three days by using small fragments (0.5-1 cm) of fresh leaves and 70% ethanol [17]. The fluid extract (*w* : *v*/g : mL) obtained was *P. sempervirens* 1 : 1.8 (55.5%).

### 2.2. Chemicals

The reagents included in standard assay packets with colorimetric and kinetic methods were obtained from BioMaxima S.A., Lublin, Poland. Thiobarbituric acid, Folin-Ciocalteu reagent, 2,2-diphenyl-1-picrylhydrazyl (DPPH), 2,2′-azino-bis(3-ethylbenzothiazoline-6-sulphonic acid) (ABTS), sulphanilamide, NEDD, VCl_3_, methanol, diethyl ether, xylenol orange, [o-cresosulfonphtalein-3,3-bis(sodium methyliminodiacetate)], *o*-dianisidine dihydrochloride (3,3′-dimethoxybenzidine), ferrous ammonium sulphate, hydrogen peroxide, sulphuric acid, hydrochloride acid, glycerol, trichloroacetic acid (TCA), and trolox were obtained from several companies (Sigma, Fluka, Merck). Standards rutin, kaempferol, and chlorogenic acid were purchased from Sigma, Germany. Ferulic acid, gallic acid, luteolin, and quercetin were obtained from Roth, Germany. All other chemicals and solvents used in the study were of analytical grade.

### 2.3. HP-MS Analysis

The phytochemical analysis was carried out using an Agilent Technologies 1100 HPLC Series system (Agilent, Santa Clara, CA, USA) assisted by mass spectrometry and using an Agilent 1100 Ion Trap SL (LC/MSD Ion Trap SL) equipped with an electrospray ion source. For the separation, a reversed-phase analytical column was employed (Zorbax SB-C18 100 × 3.0 mm i.d., 3.5 *μ*M particle). All chromatographic operations were carried out 48°C. The compounds were detected both with a UV (330 nm, 370 nm) source and in MS mode (electrospray ion source in negative mode). The chromatographic data was processed using Agilent software, ChemStation, and data analysis. The mobile phase was made up of methanol and acetic acid 0,1% (*v*/*v*). The flow rate was 1 mL/min, and the injection volume was 5 *μ*L. Based on the chromatographic conditions described above and preliminary experiments, the compounds eluted in less than 40 minutes. There were a few compounds that were not quantified due to overlapping (gentisic acid and caffeic acid), but they were identified using MS detection. The limit of quantification was 0.5 *μ*g/mL, and the limit of identification was 0.1 *μ*g/mL using the external standard method. The calibration curves (0.5-50 *μ*g/mL) range with *R* ^2^ > 0.999 linearity and detection limits between 17 and 90 ng/mL [18]. All reagents were of analytical grade [19].

### 2.4. Antioxidant Activity Assays and Total Phenolic Content

#### 2.4.1. DPPH Free Radical Scavenging Assay

Despite all the drawbacks which limit its application, scientists are still using with high interest the DPPH method, because of its easiness and rapid way in evaluating the antioxidant potential [20]. The DPPH ethanolic solution (900 *μ*M) was prepared, and 111 *μ*L was added alongside with 50 *μ*L of *Plantago* extract. The final volume (1 mL) was adjusted with a solution made of ethanol and water (1 : 1). The bleaching of DPPH was examined during 40 minutes at 517 nm, using a UV-vis spectrophotometer (Varian, Cary 50). Furthermore, percentage of unreacted DPPH was expressed in equivalents of quercetin which was used to build a calibration curve (0-30 *μ*M, *n* = 11, *R* ^2^ = 0.9905). The measurements were carried out in triplicates.

#### 2.4.2. Trolox Equivalent Antioxidant Capacity (TEAC) Assay

This method is based on the ability of natural antioxidants to scavenge the ABTS radical. In a quartz cuvette, 100 *μ*L of ABTS radical was added along with sodium acetate buffer and 10 times diluted *Plantago* sample. The final volume was 1 mL. The decreasing ABTS absorbance was monitored for 15 minutes at 420 nm, using a UV-vis spectrophotometer (Varian, Cary 50). The measurements were done in triplicates. The results were expressed as trolox equivalent (TE) *via* a calibration curve (*R* = 0.999) using trolox standard solutions.

#### 2.4.3. Determination of Total Polyphenol Content (GAE) of the Extract

The total polyphenol content of the *Plantago* extract was determined using the Folin-Ciocalteu method [21]. The extract was diluted 10 times, and 10 *μ*L was mixed with 790 *μ*L of ultrapure water, 50 *μ*L of Folin-Ciocalteu reagent, and 150 *μ*L of 20% sodium carbonate. After 30 minutes in the dark, absorbance was recorded at 750 nm, using a UV-vis spectrophotometer (Varian, Cary 50). Gallic acid (21 mg/mL) was used as standard stock solution for the calibration curve. For each replicate, the total polyphenols were determined in terms of gallic acid equivalents (GAE). The measurements were done in triplicates.

#### 2.4.4. EPR Measurements

The antioxidant reactivity was also evaluated by the kinetic profiles of the semiquinone radicals generated by alkaline treatment of the extract and monitored using electron paramagnetic resonance (EPR). The protocol for the EPR-based investigation is fully described elsewhere [22].

### 2.5. Prooxidant Activity Assays

The prooxidant reactivity of the extract was evaluated using a previously developed method that is described in detail elsewhere [23]. Briefly, the extract is treated with a catalytic amount of laccase that generates radicals from the components of the extract which are responsible for the oxidation of the ferrous oxy haemoglobin (oxyHb) into the oxidized form (metHb) which is unable to transport oxygen. The kinetic profile and the rate of the oxyHb oxidation is a marker for the reactivity of the generated radicals.

### 2.6. *In Vitro* Wound Healing Assay

#### 2.6.1. Cell Culture

Human EA.hy926 (ATCC® CRL-2922™) cells were cultivated in low-glucose Dulbecco's modified Eagle's medium (DMEM; Sigma Chemical Co., St. Louis, MO, USA) supplemented with 10% fetal bovine serum (FBS), South American origin (Gibco-Invitrogen Corp., NY, USA), and 1% antibiotic antimycotic solution (Sigma Chemical Co., St. Louis, MO, USA) at 37°C in a 5% CO_2_ atmosphere. For the experiments, cells were washed twice with a calcium- and magnesium-free buffered saline solution (CMF-BSS).

#### 2.6.2. Wound Healing Assay

A suspension containing 4 × 10^3^ cells per well were seeded in a 24-well plate and cultured for 48 h in a culture medium until confluence was reached. EA.hy926 monolayers were scratched with a 200 *μ*L pipette tip (Eppendorf, Hamburg, Germany), washed to remove debris, and cultured with a medium containing 10% FBS to facilitate cell migration. Thereafter, the cells were incubated for 6 hours with *P. sempervirens* extract. The optimal concentration employed in our experiments was 0.1 mg/mL. After 6 hours, microscope image processing was achieved using a Nikon microscope (Nikon, Tokyo, Japan). The cell migration process during the wound repair was analyzed using the image analysis software, ImageJ software (National Institutes of Health, MD). The acquired images were converted from pixels to micrometers with the use of a calibration image. For each experiment, 10 cells were randomly chosen along each edge of the wound. The data were expressed as a percentage of scratch width covered by proliferating and/or migrating cells, where the healing capacity of the untreated control cells after 10 h was set at 100% computed by *D*_*n*_ = *L*_*n*_–L0, where *D*_*n*_ and *L*_*n*_ are the net cell migration distance and the cell position at the metering point *n* (h), respectively, and L0 is the original position [24].

### 2.7. Animals

The procedures described in the present study were conducted on female Wistar rats approximately seven weeks old (150 ± 20 g). The animals were bred in the Animal Facility of Iuliu Hatieganu University of Medicine and Pharmacy, Romania, in standard conditions (12/12 light-dark cycle and humidity 30-70%, temperature 20-26°C), and with free access to standard food and water. All the experimental procedures described in the present study were in agreement with Directive 2010/63/EU and national legislation (low no. 43/2014) and were approved by the Institutional Animal Ethical Committee of the Iuliu Hatieganu University of Medicine and Pharmacy, Cluj-Napoca (22/13.12.2016). At completion of the study, rats were euthanized by cervical dislocation.

### 2.8. Study Design

The experiment was performed on Wistar adult female rats. Animals were randomly divided into 9 groups (*n* = 6): negative control group (C), inflammation group (I), groups with 7 days' pretreatment with *P. sempervirens* extract concentrations (P25, P50, and P100), and groups with 7 days' pretreatment with *P. sempervirens* extract dilutions followed by inflammation on the 7th day (IP25, IP50, and IP100). The three dilutions of the extract represent, in terms of total polyphenol content, the following values: IP25 = 40 mg GAE/kg b.w. (body weight), IP50 = 80 mg GAE/kg b.w., and IP100 = 160 mg GAE/kg b.w. The extract was administrated orally by gavage (1 mL/day), and inflammation was induced by intramuscular (i.m.) injection with turpentine oil (0.6 mL/kg b.w.). The animals were euthanized by cervical dislocation on the 8^th^ day.

### 2.9. Measurement of Oxidative Stress Parameters

The oxidative stress status was investigated using markers such as TOS, TAR, OSI, MDA, NO, CAT, and SH groups. First, serum samples were passed through 10 kDa filters and contaminant proteins were removed by extraction with 3:1 (*v* : *v*) solution of methanol : diethyl ether (1 : 9; *v* : *v*)[25]. The total oxidative status (TOS) was measured using the method proposed by [26]. The serum NO concentration was measured indirectly using the Griess reaction and expressed as nitrite *μ*mol/L [27]. The total antioxidant capacity (TAC) was determined using a colorimetric assay proposed by Erel [26]. Oxidative stress level was appreciated using the TOS/TAC ratio, known as oxidative stress index (OSI) [27]. Lipid peroxides were measured using the MDA method [28]. The main thiols in plasma were evaluated by measuring reduced glutathione (GSH). The method employed for total thiol determination was described by [28]. Catalase (CAT) activity was determined by a kinetic method, using hydrogen peroxide as substrate according to [29]. Total protein (TP), albumin (ALB), and C-reactive protein (CRP) were measured using colorimetric methods with reagents from commercial standard assay packages obtained from BioMaxima S.A. (Lublin, Poland).

### 2.10. Hematologic Analysis

Complete blood count was performed by a Coulter veterinary automatic analyzer Abacus Junior Vet (Diatron Messtechnik, Budapest, Hungary). The assay included white blood cell count (WBC), the number of neutrophils (NEU), monocytes (MON), lymphocytes (LYM), red blood cell count (RBC), haemoglobin concentration (HGB), haematocrit (HCT), mean corpuscular haemoglobin (MCH), mean corpuscular volume (MCV), mean corpuscular haemoglobin concentration (MCHC), red blood cell distribution width (RDW), platelet count (PLT), thrombocytocrit (PCT), medium platelet volume (MPV), and platelet distribution width (PDW), was performed in the Haematology Laboratory of Faculty of Veterinary Medicine from the University of Agricultural Sciences and Veterinary Medicine Cluj-Napoca.

### 2.11. Statistical Analysis

All data are reported as mean ± SEM. The Gaussian distribution was checked by the D'Agostino and Pearson omnibus normality test. One-way analysis of variance (ANOVA), followed by Bonferroni's multiple comparison test procedure, was performed. Statistical significance was set at *p* < 0.05. Statistical values and graphs were obtained using GraphPad Prism version 5.0 for Windows, GraphPad Software, San Diego, California, USA.

To examine the strengths of associations between the results, specifically Pearson correlations, we have used *Statistica 12*.0 for Windows (StatSoft Inc., USA). Multivariate data analysis was performed on the entire antioxidant and hematological parameters determined in this study using PCA (principal component analysis) incorporated in *Statistica* software.

## 3. Results

### 3.1. Phytochemical Analysis by HPLC and Phytoconstituent Profiling Using EPR Spectroscopy

**Figure 1:**
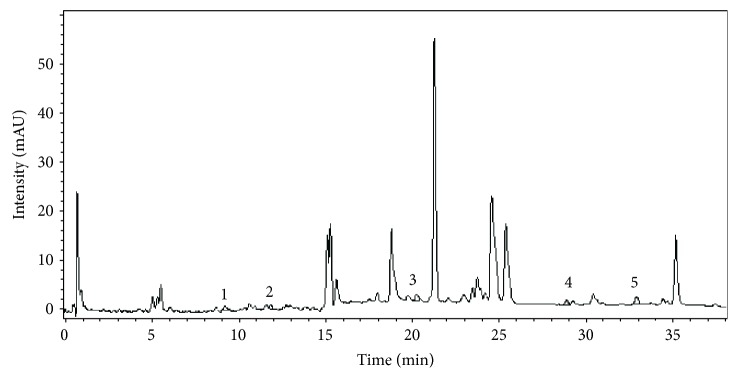
The HPLC chromatogram of the *P. sempervirens* extract ((1) p coumaric acid, (2) ferulic acid, (3) rutin, (4) luteolin, and (5) apigenin).

As shown in Table 1 and Figure 1, the chemical profile of *P. sempervirens* reveals the main compounds identified in each extract. By far, rutin, luteolin, and apigenin were the main compounds in *Plantago* samples. Therefore, we found 1,021 *μ*g/mL of luteolin, 6.48 *μ*g/mL of apigenin, and 2.34 *μ*g/mL of rutin. *P. sempervirens* by far was a rich extract because from the nine polyphenols quantified five were above the LOQ [apigenin (2.34 *μ*g/mL), rutin (2.78 *μ*g/mL), luteolin (1.02 *μ*g/mL), ferulic acid (0.45 *μ*g/mL), and p-coumaric acid (0.68 *μ*g/mL)].

In order to further analyze the major components from the chromatogram that were not identified in MS or based on retention time of the standards, UV-vis molecular spectra of these major chromatographic peaks (ca. 90% of total chromatographic area AU×min, for a 280 nm chromatogram) were exported and analyzed together with the spectra of twenty known standards using a chemometric approach. PCA analysis of the exported spectra reveals three distinct groups based on the three classes of polyphenols, i.e., benzoic acids, cinnamic acids, and flavonoids, which are in general the main phytoconstituents responsible for the antioxidant activity in plant extracts (Figure S1A—Supplementary Information). Most of the tested compounds (ca. 80% of the chromatographic area, the four most intense peaks) belong to the cinnamic acid group which present a distinct UV-vis spectrum profile. The assignment of each peak spectrum was also checked using the built-in software (Agilent ChemStation) of the HPLC chromatograph using a house-built-in data base of tens of polyphenolic standards [30]. For this purpose, a score of spectral similarity higher than 90% for any compound (with a given standard) from those three classes was an indication that the tested compound does belong to a certain class. Spectral profiles of some representatives of these classes are shown in Figure S1B—Supplementary Information.

**Figure 2:**
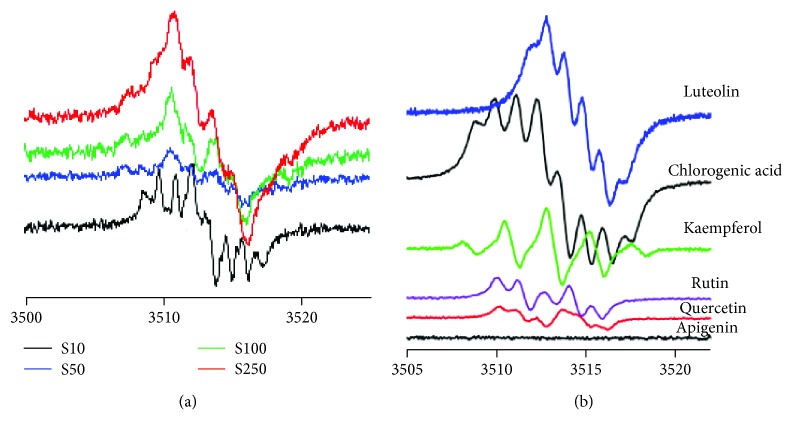
(a) *P. sempervirens*, using different dilutions of the original extract [10x (S10), 50x (S50), 100x (S100), and 250x (S250)]. (b) The spectral footprint of some constituents (luteolin, kaempferol, chlorogenic acid, rutin, quercetin, and apigenin).

A semiquinone anion radical generated fingerprint was detected using EPR measurements. As seen in Figure 2, there have been four different sample dilutions (S10, S50, S100, and S250), in order to have a clear picture about the spectra of some classes of compounds and their reactivity and chemical cross-talk of the semiquinone radicals which is concentration-dependent. Luteolin, rutin, and chlorogenic acid were some of the main compounds of interest. Apigenin, an important component in all samples, does not generate radicals, most probably due to the lack of the catechol group in the B flavonoid ring. The spectrum profile was strongly influenced by the dilution, thus 250x revealed similar shapes for both luteolin and chlorogenic acid in all samples. In the case of *P. sempervirens*, at higher concentration luteolin-based radicals dominated, whereas for lower concentrations, rutin and phenolic acids dominated. Moreover, this extract is the only one that still presents a well-defined spectrum even at 250x dilution of the original extract.

### 3.2. Antioxidant and Prooxidant Reactivity Evaluation

One of the first assessments regarding the protective potential of the extract was made in terms of *in vitro* antioxidant activity. The effectiveness of the extracts has been established in reaction with free radicals, such as DPPH and ABTS^+•^. Neutralising such radicals is directly associated with antioxidant capacity. The DPPH bleaching method indicated a 6.03 ± 0.48 mg QE/g plant of radical scavenging activity. The TEAC results measured around 459.50 ± 35.78 *μ*g TE/g. Regarding the total phenolic content using the Folin-Ciocâlteu (FC) assay, 59.14 ± 4.34 mg GAE/g plant was determined. The total phenolic content is usually strongly associated with antioxidant activity. Therefore, three different assays designate *P. sempervirens* with strong antioxidant potential. The prooxidant activity, as seen in laccase activity, was quiet low (3.15 ± 0.15 pQF).

### 3.3. *In Vitro* Wound Healing Assay

**Figure 3:**
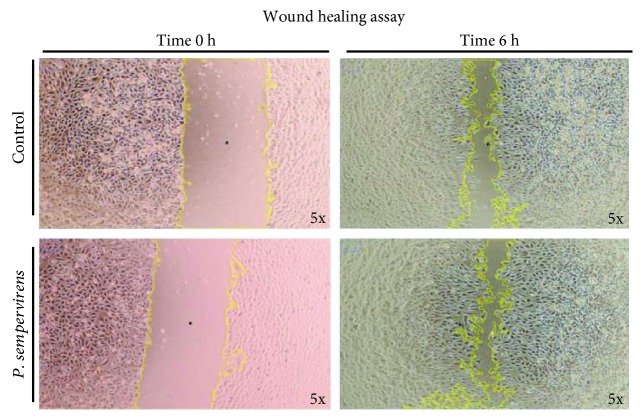
Exposure of EA.hy926 cells to *P. sempervirens* induced constant cell migration even after 6 hours.

Our results showed that the *P. sempervirens* extract presented a better wound healing response in comparison with control cells. Hence, exposure of EA.hy926 cells to *P. sempervirens* induced constant cell migration even after 6 hours (Figure 3). At this time point, the statistical analysis of cell migration revealed that the wound healing process was increased with approx. 20% for the *P. sempervirens* extract as compared to the control group (Figure 3).

#### 3.3.1. Wound Healing Assay

EA.Hy926 cells were grown to 80-90% confluence in a 6-well plate and incubated in a medium containing 1% FBS overnight. The cultured cell monolayers were scratched and cultured in DMEM supplemented with 10% FBS (control) mixed with 0.1 mg/mL plant extract. The wound closure was checked after 6 h by phase-contrast microscopy. Note the representative images of control and cells exposed to *P. sempervirens* extract after the wound scratch (time 0 h) and after the wound healing process (6 h).

### 3.4. *P. sempervirens In Vivo* Antioxidant Effect Evaluation

**Figure 4:**
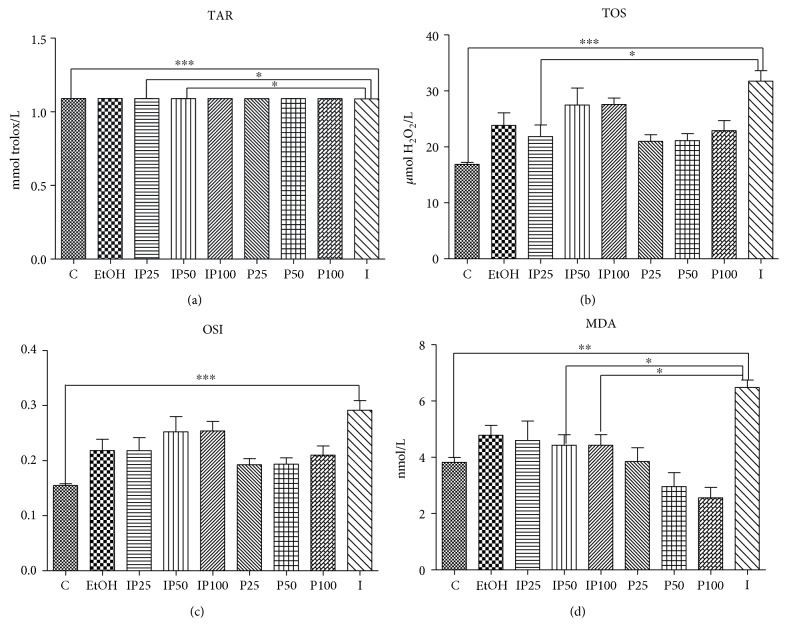
Effects of *P. sempervirens* on total antioxidant response (TAR) content (a), total oxidative stress (TOS) (b), oxidative stress index (OSI) (c), and malondialdehyde (MDA) (d) in serum of the turpentine-induced model and *P. sempervirens* extract. Data represent mean ± SEM. One-way ANOVA followed by Bonferroni's multiple-comparison test. ∗Significant at *p* < 0.05; ∗∗significant at *p* < 0.01; ∗∗∗significant at *p* < 0.00.

Experimental inflammation (as seen in group I) induced significant oxidative stress by increasing TOS (*p* < 0.001), OSI (*p* < 0.001), and MDA (*p* < 0.01) (Figure 4). The pretreatment with *P. sempervirens* afforded a significant protection (*p* < 0.05) against turpentine oil-induced inflammation, as seen in the decreased TOS concentrations in the IP25 group and the reduced MDA levels (*p* < 0.05) in the IP50 and IP100 groups. Moreover, P100 exhibited strong inhibitory effects of MDA levels, as compared with the C group (*p* < 0.05). TAR concentrations were significantly decreased during inflammation (*p* < 0.001) unlike the animals treated with the extract, which showed significant increases, as seen in the IP100 group (*p* < 0.05). OSI turned near normal after pretreatment with *P. sempervirens* (Figure 4).

Turpentine oil-induced inflammatory processes increased the level of NOx (*p* < 0.001) in correlation with TOS and OSI increased levels. As showed in Figure 4 all three *P. sempervirens* dilutions of the extract revealed significant inhibitory effects (*p* < 0.001) concerning the NO level as compared with the I group.

**Figure 5:**
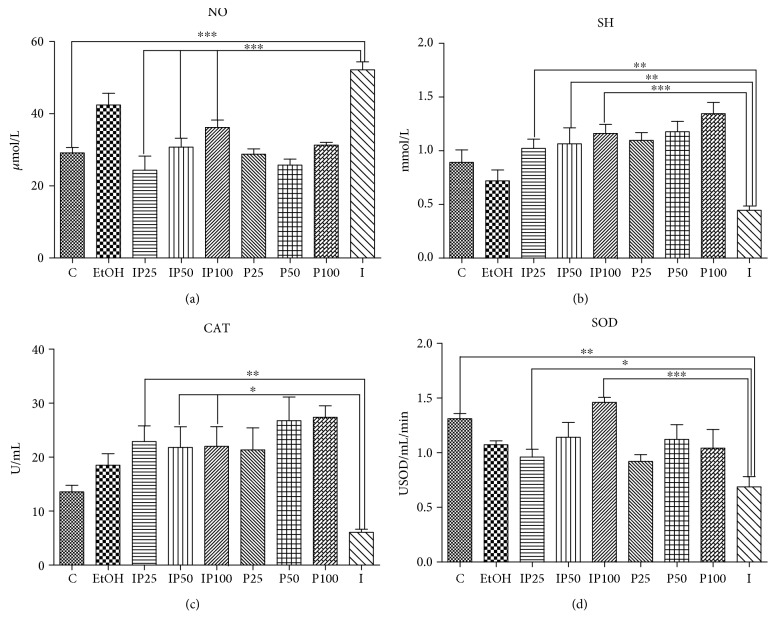
Effects of *P. sempervirens* on nitric oxide (NO) content (a), thiols (SH) (b), catalase (CAT) (c), and superoxide dismutase (SOD) (d) activity in serum of the turpentine-induced model and *P. sempervirens* extract. Data represent mean ± SEM. One-way ANOVA followed by Bonferroni's multiple comparison test; ∗Significant at *p* < 0.05; ∗∗significant at *p* < 0.01; ∗∗∗significant at *p* < 0.00.

Twenty-four hours after turpentine oil administration (I group), SOD and CAT activities were significantly compromised (*p* < 0.01) as did the concentration of SH (*p* < 0.01) as compared with the control group, as shown in Figure 5. Nevertheless, after treatment with *P. sempervirens* extract, CAT activity was markedly increased (*p* < 0.01) in IP25, and *p* < 0.05 in IP50 and IP100, as compared with the I group. SOD activity was strongly increased in IP100 (*p* < 0.001) and in IP25 (*p* < 0.05) as compared with the I group. Almost the same increase was noticed in SH concentrations (*p* < 0.01) in IP25/50 groups and in IP100 (*p* < 0.001) as shown in Figure 5. However, the P100 group increased the SH concentration, even above the C group values (*p* < 0.001). CAT activity was strongly enhanced by the extract, in both P50 and P100, whereas SOD activity was not found to be influenced by the extracts alone. EtOH, the extraction solvent, had no important effects on the investigated parameters.

### 3.5. *P. sempervirens In Vivo* Anti-inflammatory Effects

Biomarkers of inflammation such as TP, Alb, Glob, Alb/Glob, and CRP are shown in Table 2. The inflammatory effect of turpentine oil is associated with the increase in oxidative/nitrooxidative stress, as shown in previous sections, but also with the elevation of early protein metabolism biomarkers of inflammation, as seen in Table 2. The pretreatment with *P. sempervirens* had lowered the concentrations even more than control values, as shown in Table 2.

**Figure 6:**
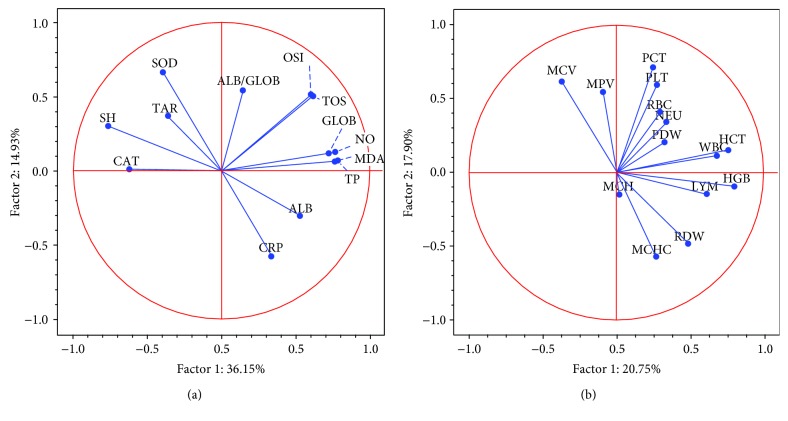
Correlation circle (loading plot) using the first two principal components of the PCA model obtained after applying PCA on (a) antioxidant parameters and (b) haematological parameters.

Our study relies on a large set of biochemical parameters, which outline a clearer picture about the biochemical status of the organism after inflammation and treatment. The obtained loading plots display the relationships between the parameters. Hence, Figure 6(a) reveals the associations of parameters according to the correlation coefficients given in Table 3. The correlation circle shows three main clusters: (1) the antioxidant parameters concentrated in the left side of the circle, (2) the oxidative stress representatives alongside with total proteins and globulins, and (3) acute-phase proteins. Table 3 confirms positive correlations between parameters, mostly between TOS-NO and NO-MDA, and very strong correlations between TOS-OSI and TP-GLOB. Taken altogether, there is a common pattern of oxidative stress strongly associated with inflammation, via NO and GLOB.

### 3.6. Hematologic Analysis

Complete blood count (Table S1—Supplementary Information) revealed not much variation among experimental groups, except WBC and NEU, which were found significantly increased in group I (*p* < 0.05) as compared with control. *P. sempervirens* in IP50 could significantly prevent the increases in WBC and NEU (*p* < 0.001). Also, IP100 significantly reduced the neutrophilia in the blood flow during inflammation (*p* < 0.01). In addition, the prevention potential of *P. sempervirens* could be proven by the P25 group, which showed a significant decrease in the WBC (*p* < 0.05). From the other haematological parameters, only for the platelets (PLT) was a small increase in group I noticed, as commonly found in systemic inflammatory responses [31].

A correlation circle of the haematological parameters indicates good relationships between parameters such as moderate correlation between MCV and MPV, very good correlation between PCT and PLT, and good correlation between HGB and HCT as well as between WBC and LYM. RDW and PDW are also well correlated, which could mean that the changes might occur simultaneously, as shown in Figure 6(b).

## 4. Discussion

Since oxidative stress is a result from an imbalance between endogenous antioxidant and oxidant production, a preventive solution against oxidative damages would be new sources of natural exogenous antioxidants with a wide range of applications [32]. Many herbs have been found to possess antioxidant properties, which should therefore minimise redox imbalances or counteract the effects of free radicals. It is well known that antioxidants could scavenge reactive species (ROS and NOS) by working as substrates for oxidative reactions, thereby blocking the chain reactions that could otherwise cause cells injuries [9].

Known for centuries, *Plantago* species are widely used in both traditional and modern medicine [4, 9]. These species have inspired many scientists for extract pharmacological evaluations such as antioxidant, anti-inflammatory, and wound healing properties [9]. This study firstly focused on a comparative phytochemical analysis and *in vitro* antioxidant and would healing effects of *P. sempervirens*. Secondly, the investigation went further for a deep exploration of the *in vivo* antioxidant and anti-inflammatory potential of the extract.

There have been many papers regarding *Plantago* species, starting with phytochemical analysis and other biological activities [9, 12, 33–39]. As stated above, the polyphenolic profile of *P. sempervirens* shared a common profile of compounds (Figure 1). The basic content of the extract is predominantly established by rutin, luteolin, and apigenin.

As shown in Results, a strong *in vitro* antioxidant activity was found for *P. sempervirens* against DPPH and ABTS radicals but also in reducing the Folin-Ciocâlteu reagent. Those results were correlated with rutin and apigenin concentrations. *P. sempervirens* was never fully described elsewhere, except for a short communication regarding the phytochemical analysis [7, 38]. Previous studies reported antioxidant activity and high contents of phenolic compounds for *P. lanceolata*, *P. cornuti*, and *P. major* [2, 3, 5, 11, 40, 41], but none of them included *P. sempervirens*.

In addition, we have been focused on the prooxidant potential of the extracts, but nonsignificant results have been found even if there are studies that suggested the possibility of luteolin, rutin, and quercetin to generate free radicals which possess prooxidant properties [23].

The cell migration process is usually initiated as a response to extracellular cues, including diffusible factors, signals on neighbouring cells, and/or signals from the extracellular matrix. The healing potential of phytomedicines is often associated with angiogenesis, which is a critical step in wound healing. Despite their traditional use in wound healing, the therapeutic value of *P. sempervirens* species has not been scientifically tested and the mechanisms are not well known. Thus, our aim was to investigate the *in vitro* potential of the *P. sempervirens* extract on the cell migration process. The influence of the tested extract on endothelial cell (EA.hy926) migration was evaluated by scratch assay/wound healing assay. According to our results obtained in this study, *P. sempervirens* extract had an increased wound repair potential for EA.hy926 cells [24]. There are several studies [42, 43] that have demonstrated the proliferative potential of *P. major* and *P. asiatica*, but as far as we know, no report was found on other species of *Plantago*. According to our results, the *P. sempervirens* extract may modulate wound healing through cell migration modulation but also *via* indirect effects such as antimicrobial, astringent, and anti-inflammatory activity. A high content of phenolic compounds such as flavonoids and phenylpropanoids, which are responsible for radical scavenging activity, may contribute to the healing process [39]. Therefore, the possible antimicrobial and anti-inflammatory activity of the *P. sempervirens* extract may have a role in the wound healing activity of these species. However, the exact mechanisms underlying these therapeutic effects of *Plantago* extracts should be elucidated in future investigations. The antioxidant function derived from *in vitro* assays confirmed the high antioxidant potential of *P. sempervirens* and supported its further use in the *in vivo* test.

Inflammation is associated with many chronic diseases such as cancer, cardiovascular disease, Alzheimer's, atherosclerosis, and other neurodegenerative diseases but also ageing [14]. The inflammatory response is closely related to oxidative and nitrooxidative stress [44]. In order to reduce/scavenge oxidants, the endogenic enzymatic and nonenzymatic antioxidants are activated. Furthermore, polyphenolic compounds were found to be efficient in the treatment of chronic inflammatory diseases associated with oxidative stress [45]. Therewith, the interest for finding exogenous sources of antioxidants, such as natural products, has significantly increased lately [46].

To further evaluate the antioxidant potential of the *P. sempervirens* extract, we have adopted an *in vivo* model of turpentine oil-induced inflammation in rats, where a nonantigenic stimulus stimulated phagocytosis as part of the cellular acute phase response [47] [14]. Such a model operated as a nonantigenic stimulus which affects the phagocytosis as part of the acute-phase cellular response associated with inflammation. Such processes activate the production of inflammatory mediators, like ROS and RNS. By reducing the concentration of inflammatory mediators, there is hope in finding the key intermediate steps in the pathway of healing a variety of disorders associated with inflammation.

Our *in vitro* data pointed *P. sempervirens* as a potential antioxidant. According to [48], flavonoids such as luteolin and apigenin had potent anti-inflammatory properties, both *in vitro* and *in vivo*. In addition, previous reports suggest that rutin acts as a preventive agent as concerns the induction and development of the innate inflammatory response, through inhibition of various proinflammatory mediators, such as NO, TNF-*α*, NF-*κ*B, and other cytokines (IL-6, IL-8) [49].

*P. sempervirens* proved to have *in vivo* antioxidant effects too by reducing the oxidants and enhancing the antioxidants. Due to high amounts of polyphenols, oxidant and antioxidant balance was clearly influenced in the antioxidant's favour, which is reflected by the investigated parameters (Figure 4). TOS depicts the level of oxidant molecules in serum which are produced endogenously and are also taken from the environment [50]. As shown in Figure 4, the lowest dose (IP25) of *P. sempervirens* had the best inhibitory effect on TOS concentrations. A consequence of the increased oxidative stress is higher lypoperoxyde formation, a process that strongly affects the cell membranes [51]. As shown in Figure 4, the MDA level was highly increased as a result of the inflammatory processes, and both *P. sempervirens* dilutions, 25 and 50, reduced MDA production. In previous experiments, MDA lowering during inflammation has been found beneficial [48, 51, 52]. All three *P. sempervirens* dilutions significantly reduced *in vivo* NO production, which could further reduce inflammation. Our findings related to NO levels were consistent with the TOS, OSI, and MDA concentrations, as seen in Table 3 and a correlation circle (Figure 6(a)).

Meanwhile, TAR levels were strongly enhanced by the extract, as seen in previous studies [53]. Our results further revealed that turpentine oil-induced inflammation could decrease the levels of nonenzymatic antioxidants, such as glutathione [14]. All three doses of *P. sempervirens* extract significantly enhanced the SH concentrations in a dose-dependent manner. Moreover, the oxidative-inflammatory reactions are strongly correlated with the activity of antioxidant enzymes [54]. There are reports that revealed correlations between the level of ROS and the decreased concentrations of antioxidants that lead to the inactivation of endogenous antioxidant enzymes, such as SOD and CAT [15, 30, 55]. In maintaining the proper physiological conditions, those endogenous enzymes play a key role by defending the cells against reactive species. SOD and CAT presented a decreased activity upon inflammation, but the *P. sempervirens* extract has proven one more time its beneficial potential by endorsing its usefulness in increasing the activity of these two enzymes with a noticeable dose-dependent influence.

In the inflammation microenvironment, some of the major players are represented by acute-phase proteins alongside the WBC. Thus, as seen in Table 2, TP and CRP concentrations were strongly influenced by the inflammation stimuli. As shown in our investigation, *P. sempervirens* demonstrated a modulator effect. An interesting finding was that the *P. sempervirens* treatment reduced acute-phase proteins not just in the inflammation groups but also even without inflammation.

The inflammation acute-phase cellular response consists of increased WBC mainly due to a higher NEU release. The *P. sempervirens* treatment revealed an immunomodulatory activity by reducing the number of WBC and NEU as well, cells known as key elements for the production of proinflammatory mediators.

In summary, our set of experiments suggests *P. sempervirens* as a valuable source of antioxidants, which acts as a preventive agent against nitro-oxidative stress.

## 5. Conclusions

The antioxidant and adjuvant potential of *P. sempervirens* was investigated using a multiple-tool palette starting with chromatography, spectroscopy, and *in vitro* and *in vivo* measurements. The antioxidant effect of the extract may be attributed to high contents of polyphenolic compounds. The *in vivo* antioxidant and anti-inflammatory effects may be related with the reduction of oxidative stress markers and early biomarkers of inflammation (WBC, NEU, CRP, and ALB) alongside the increase in activities of antioxidant enzymes, TAC, and SH concentrations. Therefore, our experiments confirmed that *P. sempervirens* would be a suitable natural remedy in inflammatory-associated pathologies.

## Figures and Tables

**Table 1:** The HPLC polyphenolic compound content determination in the studied species (*μ*g/mL extract).

Polyphenolic compounds	*P. sempervirens* (*μ*g/mL)
Gentisic acid	<0.2
Caffeic acid	<0.2
Chlorogenic acid	<0.2
*p*-Coumaric acid (1)	0.682
Ferulic acid (2)	0.456
Rutin (3)	2.787
Luteolin (4)	1.021
Apigenin (5)	2.344
Quercetin	<0.2

Note: values are in mean ± SD (*n* = 3).

**Table 2:** C-reactive protein, albumin, and total proteins of control and experimental animals.

Parameter	Control	EtOH	I	P25	P50	P100	IP25	IP50	IP100
CRP (mg/dL)	47.6 ± 6.3	50.3 ± 5.3	184.3 ± 28.4^∗^	110 ± 25.2	79.6 ± 251	162.7 ± 29.3	72.3 ± 27.5	64.3 ± 11.5	40 ± 4.3^#^
ALB (g/dL)	1.59 ± 0.3	1.79 ± 0.1	2.50±0.6^∗∗^	1.31 ± 0.9	1.70 ± 0.5	2.01 ± 0.8	1.67 ± 0.3	1.46 ± 0.4	1.16 ± 0.0^#^
TP (g/dL)	6.5 ± 0.6	9.6 ± 0.6	11.3±1.0^∗∗^	3.0 ± 0.5	4.3 ± 1.0	5.6 ± 1.1	8.5 ± 0.4	7.0 ± 0.9^#^	6.8 ± 0.6^#^
Glob (g/dL)	4.75 ± 0.6	7.58 ± 0.7	8.86 ± 0.9^∗^	1.69 ± 0.52	2.6 ± 0.9	3.67 ± 0.9	6.82 ± 0.4	5.55 ± 0.9	5.22 ± 0.4
ALB/Glob	0.36 ± 0.05	0.31 ± 0.08	0.29 ± 0.02	0.44 ± 1.08	−0.10 ± 1.03	−0.07 ± 0.50	0.25 ± 0.04	0.34 ± 0.10	0.22 ± 0.06

Values are expressed as mean ± SEM. ^∗^Significant at *p* < 0.05; ^∗∗^significant at *p* < 0.01; ^∗∗∗^significant at *p* < 0.001 (compared with control). ^#^Significant at *p* < 0.05; ^##^significant at *p* < 0.01; ^###^significant at *p* < 0.001 (compared with I).

**Table 3:** Correlation matrix (containing the correlation coefficients) between the antioxidant parameters describing the oxidative stress and the biochemical status of the system exposed to inflammation and *Plantago* treatment.

	TAR	TOS	OSI	NO	SH	CAT	SOD	MDA	TP	ALB	CRP	GLOB	ALB/GLOB	WBC	NEU
TAR	1.000	-0.215	-0.140	-0.168	**0.318**	0.200	**0.376**	**-0.309**	-0.076	-0.087	-0.285	-0.078	0.090	-0.099	0.176
TOS		1.000	**0.930**	**0.538**	-0.166	-0.337	-0.023	**0.476**	**0.278**	0.210	0.002	**0.242**	0.117	0.217	-0.122
OSI			1.000	**0.529**	-0.192	-0.239	-0.052	**0.461**	**0.285**	0.233	-0.033	**0.250**	0.120	**0.264**	-0.153
NO				1.000	-0.467	-0.463	-0.216	**0.505**	**0.497**	**0.383**	0.234	**0.441**	0.113	**0.495**	0.079
SH					1.000	**0.482**	**0.380**	-0.625	-0.584	-0.403	-0.388	-0.544	-0.075	-0.424	-0.146
CAT						1.000	0.176	-0.472	-0.379	-0.322	-0.086	-0.330	-0.044	-0.323	-0.250
SOD							1.000	-0.246	-0.164	-0.450	-0.352	-0.100	**0.252**	-0.334	0.101
MDA								1.000	**0.476**	0.245	0.106	**0.467**	0.073	**0.390**	0.187
TP									1.000	**0.382**	0.230	**0.978**	**0.253**	**0.377**	0.051
ALB										1.000	0.174	0.208	-0.129	**0.359**	0.077
CRP											1.000	0.228	-0.170	0.184	-0.118
GLOB												1.000	**0.303**	**0.339**	0.044
ALB/GLOB													1.000	0.025	0.019
WBC														1.000	0.169
NEU															1.000

[0.25-0.5]—moderate correlation; [0.5-0.75]—good correlation; [0.75-1]—very good correlation; the same for negative correlations.

## Data Availability

The data set for this study will not be publicly available until an associated PhD thesis will be published. Requests for these data sets should be directed to Anca D. Farcaș at anca.daniela.farcas@gmail.com.

## Supplementary Materials

Supplementary Materials The supplementary file contains a table (Table S1) with the hematologic parameters (complete blood count) of control and experimental animals and a figure (Figure S1) that presents the PCA analysis of the UV-vis molecular spectra of the major chromatographic peaks.
