# Supplementary material for: In Vivo Pharmacological and Anti-inflammatory Evaluation of Xerophyte Plantago sempervirens Crantz
Source: Oxid Med Cell Longev. 2019 Jun 2;2019:5049643. doi: 10.1155/2019/5049643 (PMC6589197; doi:10.1155/2019/5049643)
Supplement: Supplementary Materials — The supplementary file contains a table (Table S1) with the hematologic parameters (complete blood count) of control and experimental animals and a figure (Figure S1) that presents the PCA analysis of the UV-vis molecular spectra of the major chromatographic peaks. [file 5049643.f1.docx]

# Oxidative Medicine and Cellular Longevity - Supplementary data

***In vivo* pharmacological and anti-inflammatory evaluation of xerophyte *Plantago sempervirens*** **Crantz**

Anca D. Farcaș^1,2^, Augustin C. Moț^3^, Alina E. Pârvu^4^, Vlad Al. Toma^1,2^, Mirel A. Popa^5^, Cristina Mihai^5^, Bogdan Sevastre^6^, Ioana Roman^7^, Laurian Vlase^8^, Marcel Pârvu^1^

**Tabled S1. Hematologic parameters (Complete blood count) of control and experimental animals. Values are expressed as mean ± SEM**

| **Param.** | **Control** | **EtOH** | **I** | **P25** | **P50** | **P100** | **IP25** | **IP50** | **IP100** |
| --- | --- | --- | --- | --- | --- | --- | --- | --- | --- |
| **WBC** (10^9^/L) | 7.96±0.55 | 9.05±1.44 | 11.85±0.79^***^ | 7.72±0.60 | 7.49±0.82 | 7.35±0.94 | 8.96±1.12 | 5.32±1.29^##^ | 7.44±0.59 |
| **LYM** (10^9^/L) | 3.52±0.33 | 5.05±0.60 | 4.02±0.43 | 5.02±0.54 | 4.57±0.60 | 4.74±0.80 | 4.61±0.64 | 3.22±0.74 | 3.79±0.46 |
| **MON** (10^9^/L) | 0.59±0.33 | 0.71±0.32 | 0.52±0.15 | 0.34±0.15 | 0.19±0.15 | 0.29±0.19 | 0.76±0.21 | 0.44±0.14 | 0.70±0.15 |
| **NEU** (10^9^/L) | 3.84±0.52 | 3.08±0.91 | 6.15±0.43 | 2.35±0.27 | 2.73±0.30 | 2.31±0.40 | 3.58±0.57^#^ | 1.65±0.52^###^ | 2.94±0.32^##^ |
| **RBC**(10^12^/L) | 8.46±0.30 | 9.25±0.38 | 8.67±0.28 | 8.82±0.50 | 8.44±0.50 | 8.59±0.24 | 9.74±0.80 | 7.70±0.86 | 8.67±0.47 |
| **HGB** (g/L) | 15.20±0.48 | 15.60±0.59 | 14.22±0.55 | 15.12±0.57 | 15.02±0.41 | 14.65±0.22 | 17.03±1.38 | 12.82±1.55 | 14.38±0.69 |
| **HCT** (%) | 50.68±1.59 | 51.81±1.97 | 50.56±2.05 | 48.96±2.17 | 48.14±1.51 | 48.48±1.38 | 55.87±4.52 | 44.70±4.96 | 49.99±2.22 |
| **MCV** (fL) | 57.67±1.11 | 55.83±0.30 | 58.33±1.22 | 55.83±0.94 | 56.83±0.87 | 56.50±0.56 | 57.33±0.84 | 58.17±1.24 | 57.83±1.32 |
| **MCH** (pg) | 17.28±0.37 | 16.87±0.19 | 16.37±0.28 | 17.32±0.86 | 17.78±0.36 | 17.08±0.24 | 17.50±0.06 | 16.50±0.42 | 16.62±0.35 |
| **MCHC** (g/L) | 30.03±0.57 | 3.10±0.25 | 28.13±0.23 | 31.13±1.47 | 31.23±0.69 | 30.30±0.43 | 30.53±0.39 | 28.47±0.64 | 28.78±0.29 |
| **RDWs** (fL) | 17.75±0.24 | 17.85±0.60 | 16.77±0.43 | 17.15±0.39 | 17.22±0.47 | 17.45±0.23 | 17.25±0.28 | 16.58±0.26 | 16.63±0.30 |
| **PLT** (10^9^/L) | 1090±38.82 | 1084±76.79 | 1204±68.70 | 1126±44.15 | 999.15±57.66 | 1126±78.86 | 1021±102.2 | 928.3±118.4 | 1061±39.46 |
| **PCT** (%) | 0.84±0.04 | 0.77±0.06 | 0.83±0.05 | 0.79±0.02 | 0.74±0.04 | 0.81±0.04 | 0.76±0.08 | 0.69±0.08 | 0.78±0.03 |
| **MPV** (fL) | 7.48±0.11 | 7.05±0.12 | 7.48±0.12 | 17.08±9.98 | 7.43±0.15 | 7.23±0.17 | 7.48±0.15 | 7.45±0.03 | 7.38±0.07 |
| **PDWs** (fL) | 34.15±0.32 | 33.60±0.32 | 33.87±0.34 | 32.92±0.37 | 33.53±0.24 | 33.33±0.40 | 34.33±0.30 | 33.40±0.14 | 33.20±0.28 |

* Significant at *p* < 0.05; ** Significant at *p* < 0.01; *** Significant at *p* < 0.001 (compared with Control)

^#^ Significant at *p* < 0.05; ** Significant at *p* < 0.01; *** Significant at *p* < 0.001 (compared with I)


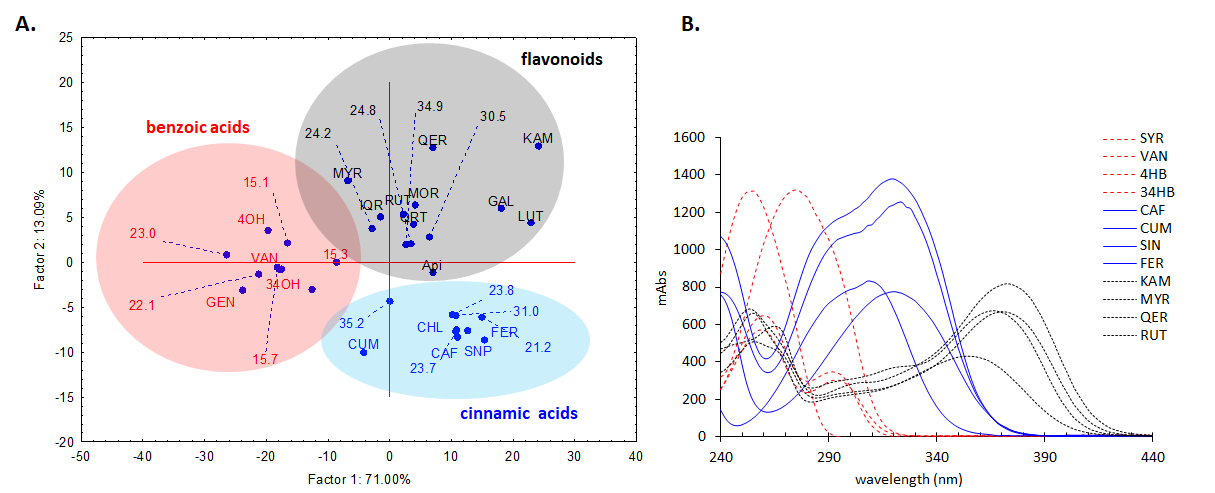


**Figure S1. A.** *PCA analysis of the DAD UV-Vis molecular spectra of the main unidentified chromatographic peak from the* Plantago sempervirens *extract (indicated with their chromatographic elution time), together with the known standards, showing their grouping in three distinct classes of polyphenols, i.e. benzoic acids (red), cinnamic acids (blue) and flavonoids (black). Standards: 4OH-4-hidroxybenzoic acid, 34OH-3,4-dihydroxybenzoic acid, API-apigenin, CAF-cafeic acid, CHL-chlorogenic acid, FER-ferulic acid, GAL-galangin, GEN-gentisic acid, IQR-isoquercitrin, KAM-kaempferol, LUT-luteolin, MOR-morin, MYR-myricetin, CUM-p-coumaric acid, QER-quercetin, QRT-quercitrin, RUT-rutin, SNP-sinapic acid, SYR-syringic acid, VAN-vanilic acid* ***B.*** *Molecular DAD UV-vis spectra of some representative standard compounds from the three polyphenolic classes.*
